# Capsaicin induces apoptosis in PC12 cells through ER stress

**DOI:** 10.3892/or.2013.2921

**Published:** 2013-12-13

**Authors:** OLGA KRIZANOVA, IVETA STELIAROVA, LUCIA CSADEROVA, MICHAL PASTOREK, SONA HUDECOVA

**Affiliations:** 1 Institute of Molecular Physiology and Genetics, Slovak Academy of Sciences, 833 34 Bratislava, Slovak Republic; 2 Molecular Medicine Center, Slovak Academy of Sciences, 831 01 Bratislava, Slovak Republic; 3 Cancer Research Institute, Slovak Academy of Sciences, 833 91 Bratislava, Slovak Republic

**Keywords:** capsaicin, ER stress, apoptosis, PC12 tumor cells

## Abstract

Capsaicin, the pungent agent in chili peppers, has been shown to act as a tumor-suppressor in cancer. In our previous study, capsaicin was shown to induce apoptosis in the rat pheochromocytoma cell line (PC12 cells). Thus, the aim of the present study was to determine the potential mechanism by which capsaicin induces apoptosis. We treated PC12 cells with 50, 100 and 500 μM capsaicin and measured the reticular calcium content and expression of the reticular calcium transport systems. These results were correlated with endoplasmic reticulum (ER) stress markers CHOP, ATF4 and X-box binding protein 1 (XBP1), as well as with apoptosis induction. We observed that capsaicin decreased reticular calcium in a concentration-dependent manner. Simultaneously, expression levels of the sarco/endoplasmic reticulum pump and ryanodin receptor of type 2 were modified. These changes were accompanied by increased ER stress, as documented by increased stress markers. Thus, from these results we propose that in PC12 cells capsaicin induces apoptosis through increased ER stress.

## Introduction

Endoplasmic reticulum (ER) stress (ERSR) is engaged in many cellular functions such as protein synthesis, folding and storage, as well as in calcium signaling. When disturbances in these functions occur and levels of misfolded proteins in the ER increase, the goal of ERSR is to enhance protein folding, reduce new protein synthesis and clear misfolded proteins. In the case of a default in this function, ERSR may trigger apoptotic cell death for elimination of defective cells (1). There is much evidence that enhanced ERSR, triggering apoptosis, is associated with many degenerative diseases (reviewed in ref 2). An opposite situation may be observed in tumor cells, which are generally resistant to apoptotic stimuli. For example, malignant glioma cells are in a condition of constant low ERSR, which possibly contributes to their resistance to treatment (1). PC12 cells, derived from rat pheochromocytoma, possess several mechanisms enabling them to counteract apoptosis. Recently, a pathway mediated by heat shock proteins, which prevents ERSR-induced apoptosis has been revealed (3). Chaperone protein HSP72 enhances IRE1a-XBP1 signaling through a physical interaction (4), and HSP90 is a key protein in several pathways of cell proliferation and tumor progression (3,5). Resistance to apoptosis is frequently mediated by overexpression of anti-apoptotic or the absence of pro-apoptotic proteins from the Bcl-2 family (6,7). This pathway has been studied during the last decade of research, and several substances modulating either expression or activity of these proteins have been tested as new potential anticancer drugs (6). Saito *et al* (8) induced apoptosis in PC12 cells by multitargeted receptor tyrosine kinase inhibitor (sunitib), which modulated the Bcl-2 and BAD pathways. The cytotoxic effect of this drug was pronounced when autophagy in these cells was inhibited (9).

A special group of potential anticancer drugs include active substances from traditional medicinal plants. Extracts from plants of the *Phyllanthus* species were shown to interfere with multiple signaling cascades in human prostate carcinoma PC-3 cells and were able to trigger apoptotic cell death (10). Triptolide isolated from the plant *Trypterygium wilfordii* was shown to inhibit proliferation of a variety of cancer cells acting through the NF-κB cascade (11,12). Several investigators have reported the ability of capsaicin (*Capsicum* species) to prevent tumorigenesis by triggering apoptotic pathways. Capsaicin (8-methyl-N-vanillyl-6-nonenamide), a member of the vanilloid family, binds to a receptor called the vanilloid receptor subtype 1 (TRPV1), which has been shown to be a member of the superfamily of TRP ion channels and permits cations to pass through the cell membrane and into the cell when activated. Ito *et al* (13) showed that capsaicin induces apoptosis in leukemic cells through oxidative stress. Experiments more focused on receptor TRPV1 have shown its anti-oncogenic effects in transitional urothelial cancer of the human bladder. A progressive decrease in TRPV1 expression during the transitional stage of cancer was found to trigger the development of a more aggressive phenotype and invasiveness (14). When capsaicin was applied to TRPV1-knockout urothelial cancer cells, an even more aggressive form of tumor was observed (15). It was also recently shown that the TRPV1 channel activated by capsaicin caused an increase in intracellular calcium concentrations in mammalian skeletal muscle (16).

In our previous studies we proved that two herbal compounds, triptolide (TTL) and capsaicin (Caps), are inhibitors of the nuclear transcription factor NF-κB in PC12 and MPC cells (11). Inhibition of this factor caused enhanced expression of norepinephrine transporter (NET) and apoptosis (11). The aim of the present study was to evaluate the mechanisms triggered by capsaicin leading to the apoptosis in PC12 cells. We focused on primary signals, which may be connected with calcium homeostasis and calcium transporting proteins in the membrane of the endoplasmic reticulum, as well as on typical factors of ERSR and apoptosis.

## Materials and methods

### Cell cultivation and treatment

PC12 cells (German Collection of Microorganisms and Cell cultures, DSMZ, Braunschweig, Germany) derived from rat pheochromocytoma were cultured in Dulbecco’s minimal essential medium (Biochrom AG, Berlin, Germany) with high glucose (4.5 g/l) supplemented with 15% fetal calf serum and penicillin and streptomycin antibiotics. Cells were cultured in a water-saturated atmosphere at 37°C with 5% CO_2_. Treatment was performed by adding of 50, 100 and 500 μM (E)-capsaicin (Caps; Merck, Germany) directly to the cultivation media for 24 h.

### RNA isolation and relative quantification of mRNA levels by RT-PCR and qPCR

Total RNA was isolated by TRI reagent (MRC Ltd., Cincinnati, OH, USA). Briefly, cells were scraped and homogenized by a pipette tip in sterile water and afterwards TRI reagent was added. After 5 min the homogenate was extracted by chloroform. RNAs in the aqueous phase were precipitated by isopropanol. RNA pellet was washed with 75% ethanol and stored in 96% ethanol at −70°C. The purity, quantity and integrity of isolated RNAs were assessed using GeneQuant Pro spectrophotometer (Amersham Biosciences, Buckinghamshire, UK). Reverse transcription was performed using 1.5 μg of total RNAs and Ready-To-Go You-Prime First-Strand beads with pd(N)6 primer (both from GE Healthcare Life Sciences, USA). PCR specific for the unspliced form of X-box binding protein 1 (XBP1) (GI: 51259532) was performed with primers: XBP1 forward, 5′-AGCGCTGCCGCTCATGCTTC-3′ and reverse, 5′-TCTCGCGCAGTCTGTGCTGC-3′; for ATF4 (GI: 165971604) forward, 5′-GGCCACCATGGCGTATTAAGA-3′ and reverse, 5′-GACATTAAGTCCCCCGGCCAA-3′; and for CHOP (GI: 2660765) forward, 5′-AGGGCTAGCTTGGTCCTAGA-3′ and reverse, 5′-CCCCAAGTCCTGAACTCCAC-3′. For anti-apoptotic Bcl-2 (GI: 408946) primers were: Bcl-2 forward, 5′-ACTTCTCTCGTCGCTACCGT-3′ and reverse, 5′-GTTCCACAAAGGCATCCCAG-3′; for Bax (GI: 3320116) forward, 5′-GAAGCTGAGCGAGTCTCTCC-3′ and reverse, 5′-GATCAGCTCGGGCACTTTAG-3′; for SERCA2 (GI: 8392934) forward, 5′-ATTGTTCGAAGTCTGCCTTCTGTG-3′ and reverse, 5′-CATAGGTTGATCCAGTATGGTAAA-3′. Primers for rat IP3R1 (GI: 1055286) were: IP3R1 forward, 5′-GTGGAGGTTTCATCTGCAAGC-3′ and reverse, 5′-GCTTTCGTGGAATACTCGGTC-3′; for IP3R2 (GI: 13752805) IP3R2 forward, 5′-GCTCTTGTCCCTGACATTG-3′ and reverse, 5′-CCCATGTCTCCATTCTCATAGC-3′; and for IP3R3 (GI: 6981109) forward, 5′-CTGCCCAAGAGGAGGAGGAAG-3′ and reverse, 5′-GAACAGCGCGGCAATGGAGAAG-3′. Primers for ryanodine receptor type 2 (RyR2) (GI: 2305245) were forward, 5′-CATCGGTGAAATTGAAGA-3′ and reverse, 5′-AGCATCAATGATCAAACCTTG-3′. As housekeeping genes rat β-actin A (GI: 42475962) was used with primers BA forward, 5′-AGTGTGACGTTGACATCCGT-3′ and reverse, 5′-GACTGATCGTACTCCTGCTT-3′ or cyclophilin (GI: 203701) with primers CYCLO forward, 5′-CGTGCTCTGAGCACTGGGGAGAAA-3′ and reverse, 5′-CATGCCTTCTTTCACCTTCCCAAAGAC-3′. The same primers were used for RT-PCR and also for real-time quantitative PCR. Products of RT-PCR were analyzed on a 2% agarose gel and signals were evaluated by PCBAS 2.0 software. Real-time PCR was performed on PikoReal 96 cycler with DyNAmo Color Flash SYBR-Green Master Mix (both from Thermo Fisher Scientific, Hampshire, UK). Results were evaluated by PikoReal software 2 as a peak area for every well and quantified relatively from Cq values according to the formula ΔΔCq = ΔC_q(sample)_ − ΔC_q(housekeeper)_, where the rat β-actin A was used as the housekeeping gene.

### [Ca^2+^]_free_ measurement in the reticular fraction with Rhod-5N dye

We used the method as was described in our previous study (17). Briefly, cells were scraped from wells, sedimented by centrifugation and washed with phosphate-buffered saline (PBS) solution. Gentle lysis was performed with 100 μl of cell lysis buffer provided in the kit for cytoplasmic and nuclear protein isolation (ProteoJet™; Fermentas, Germany) in the presence of dithiothreitol (DTT) (10 mM). Post-mitochondrial fractions with ER cisternae were isolated as described in a study by Kalén *et al* (18). Pellets from the post-mitochondrial fraction were homogenized in nuclear lysis buffer from the ProteoJet™ kit and pipetted to wells in a 24-well plate. To each sample, Rhod-5N fluorescent dye was added to a final concentration of 20 μM. Measurements were captured using the BioTek (BioTek, Germany) fluorescence reader (excitation, 551 nm/emission, 576 nm). The fluorescent (F) signal was saturated by adding EGTA solution, pH 7.0, to the final concentration of 2.5 mM (Fmin). Fmax value was measured by adding CaCl_2_ to the final concentration of 0.5 mM. Final values of [Ca^2+^]_free_ were calculated according to the formula: [Ca^2+^]_free_ = Kd [(Fmax − F)/(F − Fmin)], where Kd for Rhod-5N is 320 nM. Results were calculated relative to protein levels, which were determined in the samples by the method of Lowry *et al* (19).

### Cytofluorometric analysis of the mitochondrial membrane potential

Analysis of mitochondrial membrane potential via Ψ_m_ was performed as previously described (20). Briefly, cells were collected by centrifugation at 1000 × g for 5 min and washed twice with cold PBS. Incubation was performed in 200 μl of PBS/0.2% BSA containing 4 μM JC-1 fluorescent dye (5,5′,6,6′-tetrachloro-1,1′,3,3′-tetraethylbenzimidazolyl carbocyanine iodide) and 7-aminoactinomycin D (7-AAD) (5 ng/μl) both from Invitrogen Life Technologies, USA for 30 min at 37°C in the dark. 7-AAD was used to exclude the population of necrotic cells. Cell data were acquired using the EPICS Altra (Beckman Coulter) flow cytometer equipped with a 488-nm excitation laser, and fluorescence emission of JC-1 green, JC-1 red, and 7-AAD was measured using a band pass filter set at 525, 575 and 675 nm, respectively. Forward and side light scattering characteristics were used to exclude cell debris from the analysis. For each analysis, 1×10^4^ cells were acquired, and the ratio of JC-1 red/JC-1 green fluorescence of viable cells (7-AAD negative) was used to calculate the decrease in mitochondrial membrane potential (ΔΨ_m_). Data were analyzed by FCS4 software (De Novo Software, Los Angeles, CA, USA).

### Detection of apoptosis with Annexin-V-FLUOS

Cells were sedimented and washed with 1Χ PBS solution. The cell pellet from each well was labeled using the Annexin V-FLUOS/propidium iodide labeling kit (Roche Diagnostics, Germany) according to the protocol of the producer and incubated at room temperature in the dark for 20 min. After the incubation was completed, the reaction was halted by adding three volumes of ice cold PBS. Measurements were performed on an Accuri C6 flow cytometer, and results were evaluated by the C-Flow sampler 2.1 (BD Accuri Cytometers, Ann Arbor, MI, USA).

### Western blot analysis

Cells were scraped and suspended in 10 mM Tris-HCl, pH 7.5, 1 mM phenylmethylsulfonyl fluoride (PMSF) (Serva, Germany), protease inhibitor cocktail tablets (Complete EDTA-Free; Roche Diagnostics) and subjected to centrifugation for 10 min at 10,000 × g at 4°C. The pellet was resuspended in Tris-buffered saline (TBS) containing 50 μM CHAPS [3[(3-cholamidopropyl)dimethylammonio]-l-propanesulfonate)] (Sigma, USA) and then incubated for 10 min at 4°C. The lysate was centrifuged for 10 min at 10,000 × g at 4°C. Protein concentration of the supernatants was determined by the method of Lowry *et al* (19). Protein extract (20 μg) from each sample was separated by electrophoresis on 10% SDS polyacrylamide gels and proteins were transferred to the Hybond-P membrane using semi-dry blotting (Owl, Inc., Portsmouth, NH, USA). Membranes were blocked in 5% non-fat dry milk in TBS with Tween-20 (TBS-T) overnight at 4°C and then incubated for 1 h with XBP1/TREB5 monoclonal antibody (Antibodies-online GmbH, Aachen, Germany), CREB2/ATF4 (sc-200) polyclonal antibody, GADD153/CHOP (sc-575) polyclonal antibody, Bax (sc-7480) polyclonal antibody (all from Santa Cruz Biotechnology, Inc., Santa Cruz, CA, USA) and the Bcl-2 polyclonal antibody (Abcam, Cambridge, UK). Following washing, membranes were incubated with secondary antibodies to rabbit and mouse IgG-conjugated to horseradish peroxidase for 1 h at room temperature. For relative quantification, each membrane was reprobed for housekeeper GAPDH mouse monoclonal antibody (Abcam). An enhanced chemiluminiscence detection system (ECL Plus; Amersham Biosciences) was used to detect the bound antibody. The optical density of individual bands was quantified using PCBAS 2.0 software.

### Immunofluorescence

For immunofluorescence, cells were fixed with ice-cold methanol. In these experiments, the same antibodies against XBP1, ATF4 and CHOP were used as in western blot analysis. Cells grown on glass coverslips were fixed in ice-cold methanol at −20°C for 5 min. Non-specific binding was blocked by incubation with PBS containing 3% BSA for 60 min at 37°C. Cells were then incubated with primary antibody diluted 1:500 in PBS with 1% BSA (PBS-BSA) for 1 h at 37°C, washed three times with PBS-BSA for 10 min, incubated with CF Fluor^®^ 488 goat anti-rabbit IgG (Biotium, Hayward, CA, USA) diluted 1:1,000 in PBS-BSA for 1 h at 37°C and washed as previously described. Finally, cells were mounted onto slides in mounting medium with CitiFluor (Agar Scientific Ltd., Essex, UK), and analyzed using the LSM 510 Meta microscope (Carl Zeiss, Jena, Germany) with EC Plan-Neofluar ×40 objective. Cellular nuclei were stained with 4′,6-diamidino-2-phenylindole (DAPI) (Life Technologies Carlsbad, CA, USA). All images were captured using the same camera and microscope.

### Statistical analysis

Each value was the average of 24-wells in at least 4 independent cultivations of PC12 cells. Results are presented as means ± SEM. Statistical differences between the groups were determined by ANOVA. p<0.05 was considered to indicate a statistical significant result. An adjusted t-test with p-values corrected by the Bonferroni’s method was used for multiple comparisons (Instat; GraphPad Software).

## Results

**Figure 1:**
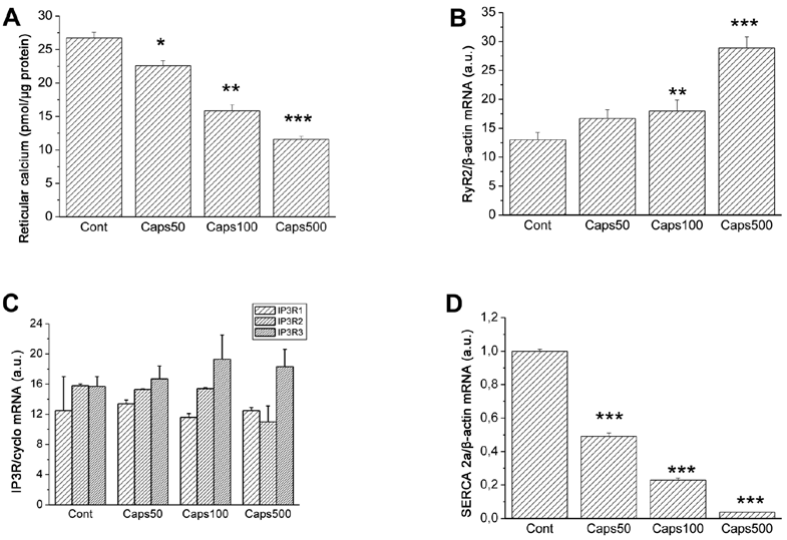
(A) Capsaicin induces a significant release of calcium from the reticular fraction of PC12 cells. The extent of this depletion was concentration-dependent, and the most pronounced effect was noted in cells treated with 100 and 500 μM capsaicin. The decrease in calcium content was accompanied by (B) increase in RyR2 calcium release channel expression and (D) decrease in sarco-endoplasmic ATPase (SERCA2) expression. In contrast to RyR2, expression of SERCA2 was affected at 50 μM capsaicin. (C) Expression of IP3 receptors was not significantly altered. Results are presented as means ± SEM and represent an average of triplicate samples from 3 cultivation. Statistical significance when compared with controls ^*^p<0.05, ^**^p<0.01 and ^***^p<0.001. RyR2, ryanodine receptor type 2.

Capsaicin (Caps) modulated the calcium turnover in the reticular fraction of PC12 cells in a concentration-dependent manner. Whereas 50 μM Caps moderately lowered the calcium content when compared to the control, 100 and 500 μM Caps caused a substantial and significant decrease (Fig. 1A) [from 26.74±0.82 (control) to 15.86±0.86 and 11.60±0.43 pmol/μg of protein]. This decrease was accompanied by changes in the expression of reticular calcium transport systems. Expression of calcium release channel RyR2 was elevated mainly at 100 and 500 μM Caps [Fig. 1B; from 13.0±1.3 (control) to 18.0±1.9 and 28.9±2.8 a.u., respectively]. In contrast, expression of SERCA2, which is responsible for transport of calcium to endoplasmic reticulum, was significantly reduced at all three Caps concentrations tested [Fig. 1D; from 1.0 (control) to 0.038 a.u. (at 500 μM Caps)]. Notably, no significant effect by Caps was noted on the expression of other calcium release channels, IP3 receptors (IP3R)1, 2 and 3 (Fig. 1C).

**Figure 2:**
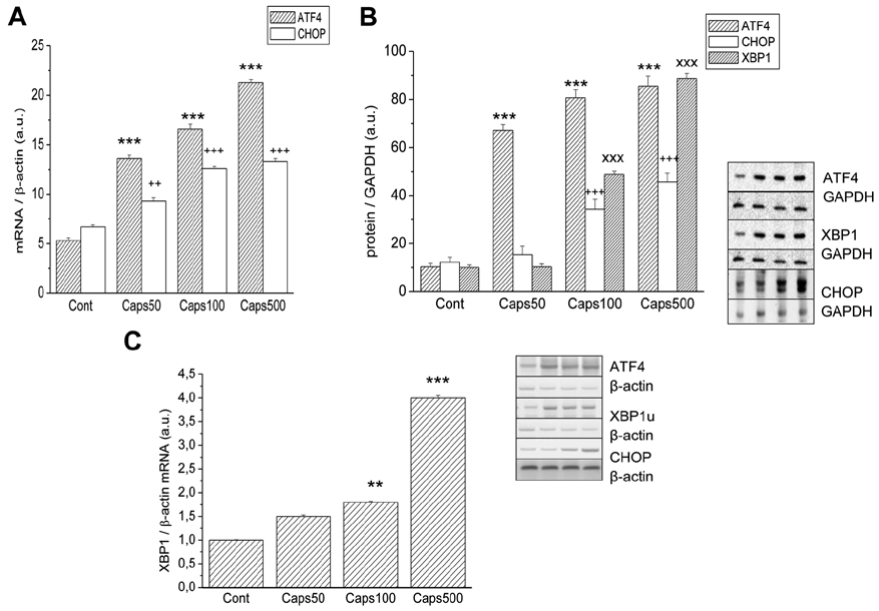
(A) mRNA signals for ATF4 and CHOP, obtained by RT-PCR, were significantly elevated by capsaicin in a dose-dependent manner. (C) Relative quantification of XBP1 transcription factor mRNA by real-time PCR showed elevated levels at 100 and 500 μM Caps. Treatment with 500 μM Caps caused a 4-fold elevated expression of XBP1 mRNA. Rat β-actin was used as a housekeeping gene. (B) Increased mRNA signals for all 3 ER stress markers were accompanied by an increase in their protein signals as detected by western blotting. Results are presented as means ± SEM and represent an average of triplicate samples from 3 cultivation. Statistical significance when compared with controls for ATF4; ^*^p<0.05, ^**^p<0.01 and ^***^p<0.001. Statistical significance when compared with controls for CHOP; ^++^p<0.01 and ^+++^p<0.001. Statistical significance when compared with controls for XBP1; ^xxx^p<0.001. XBP1, X-box binding protein 1.

**Figure 3:**
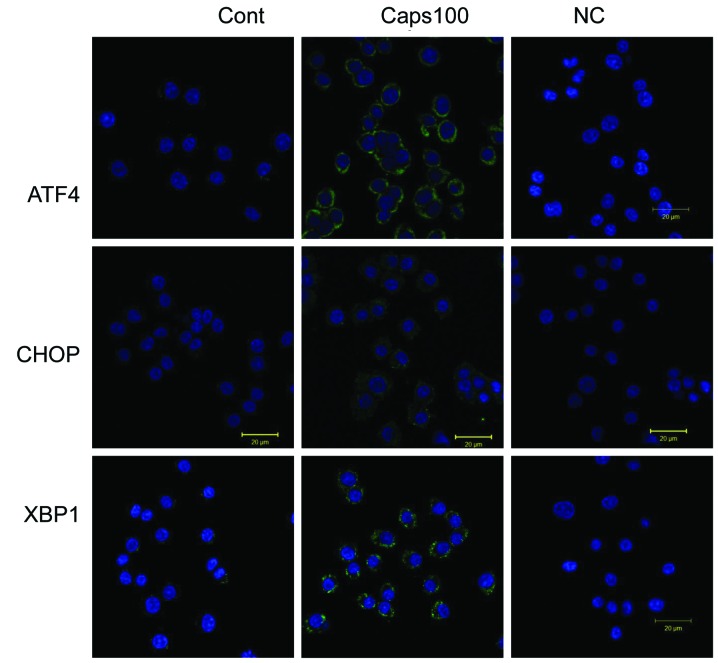
Immunofluorescent signals of the expression of ER stress markers ATF4, CHOP and unspliced XBP1 in PC12 cells exposed to capsaicin. Cont, untreated cells; Caps100, cells-treated for 24 h with 100 μM capsaicin; Nc, negative control, for which cells were processed with secondary antibody only. Treatment with 100 μM capsaicin induced elevated expression of all 3 markers in the endoplasmic reticulum (green stain). Cellular nuclei were stained with DAPI (blue stain). ER, endoplasmic reticulum; XBP1, X-box binding protein 1.

Gene and protein expression of the ER stress markers ATF4, CHOP and XBP1 also exhibited a concentration-dependence on capsaicin. Significantly elevated mRNA signals were noted for ATF4 and CHOP (Fig. 2A) and XBP1 (Fig. 2C). CHOP mRNA was significantly elevated at 100 and 500 μM Caps [from 6.7±0.2 (control) to 12.6±0.21 and 13.3±0.3 a.u., respectively]. A different pattern of expression was observed for ER stress marker ATF4, where the mRNA signal was elevated all three capsaicin concentrations (50, 100, 500 μM) [from 5.3±0.3 (control) to 13.6±0.33; 16.6±0.5 and 21.3±0.3 a.u., respectively]. Relative quantification of the unspliced form of the XBP1 by real-time PCR revealed its mRNA elevation at 100 and 500 μM Caps. Particularly, 500 μM Caps caused a 4-fold elevated expression of XBP1 (Fig. 2C). An increase in mRNA signals was accompanied by an increase in protein signals as determined by western blotting (Fig. 2B). Levels of CHOP and XBP1 proteins were increased significantly at 100 and 500 μM Caps [CHOP: 12.2±2.1 (control) to 34.3±4.1 and 45.6±3.8 a.u.; XBP1: from 10.0±1.1 (control) to 48.9±1.3 and 88.7±2.1 a.u., respectively]. ATF4 protein signal was elevated at all three concentrations of capsaicin [from 10.3±1.4 (control) to 67.2±2.4; 80.7±3.3 and 85.6±4.1 a.u., respectively]. A strong increase in immunofluorescent signals at the ER for ATF4, CHOP and unspliced XBP1 were observed in the PC12 cells treated with 100 μM Caps at 24 h (Fig. 3).

**Figure 4:**
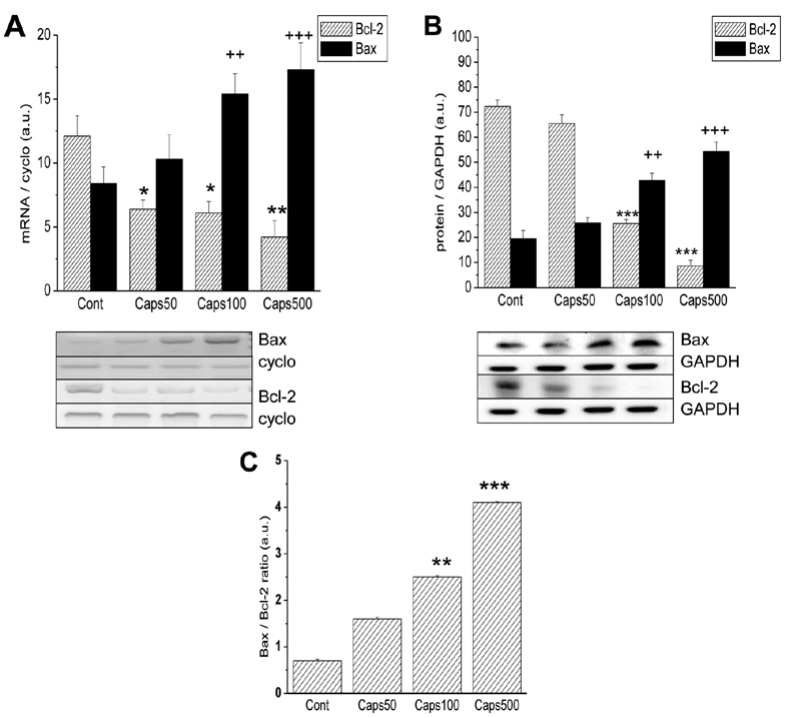
Effect of capsaicin on expression of pro-apoptotic and anti-apoptotic proteins Bax and Bcl-2 in PC12 cells. (A) Signals obtained by RT-PCR indicate a significant decrease in anti-apoptotic protein Bcl-2 mRNA (stripped bar) in contrast to pro-apoptotic protein Bax, where significant increase is noted (solid bar). As a housekeeping gene for relative quantification cyclophilin was used. (C) Ratio of mRNA Bax/Bcl-2 signals indicates an increase in the pro-apoptotic signal in cells. (B) Results obtained by western blot analysis were similar to RNA. Bcl-2 protein was decreased (stripped bar) and Bax protein levels (solid bar) were increased with higher concentrations of capsaicin applied to cells. Results are presented as means ± SEM and represent an average of triplicate samples from 3 cultivation. Significance for Bcl-2 was ^*^p<0.05; ^**^p<0.01 and ^***^p<0.001 and for Bax ^++^p<0.01 and ^+++^p<0.001.

Decreased reticular calcium content is associated mainly with changes in the expression and function of calcium rheostat proteins from the Bcl-2 family. We monitored two typical members of this family, Bcl-2 known as an anti-apoptotic protein and Bax as a representative pro-apoptotic protein acting on the ER and mitochondria. Fig. 4 shows that 100 and 500 μM capsaicin caused a significant elevation in mRNA and protein levels of pro-apoptotic Bax [Fig. 4A; from 8.4±1.3 (control) to 15.4±2.1 a.u. (at 100 μM) and Fig. 4B from 19.54±3.3 (control) to 42.74±2.9 and 54.34±3.8 a.u. (at 100 and 500 μM, respectively)]. In contrast, levels of anti-apoptotic Bcl-2 were dramatically decreased at both the mRNA [Fig. 4A; from 12.1±1.6 (control) to 6.4±0.7 6.1±0.9 and 4.2±1.3 a.u. (at 50, 100 and 500 μM, respectively)] and protein levels [Fig. 4B; from 72.34±2.6 (control) to 25.64±1.6 and 8.64±2.3 a.u. (at 100 and 500 μM, respectively)] This effect resulted in a change in the ratio of Bax/Bcl-2 content, which was shifted in favor of Bax (Fig. 4C).

**Figure 5:**
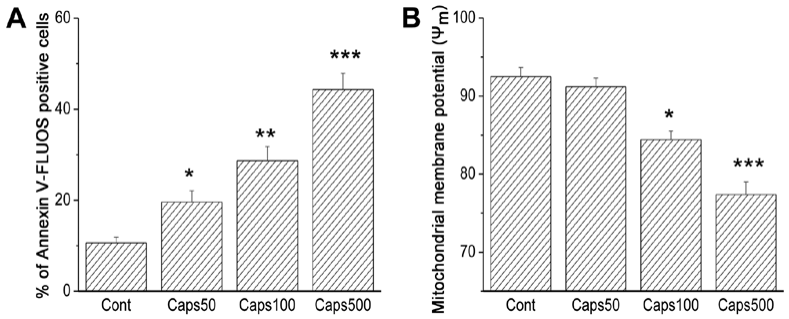
Apoptosis in PC12 cells following treatment with capsaicin. (A) Increased population of cells binding with Annexin V-FLUOS are noted after 24 h of treatment with capsaicin (^*^p<0.05; ^**^p<0.01 and ^***^p<0.001). (B) Decrease in of mitochondrial membrane potential was significant at 100 and 500 μM capsaicin (^*^p<0.05 and ^***^p<0.001).

Possible induction of apoptosis by capsaicin in PC12 cells was tested by a decline in mitochondrial membrane potential ΔΨ_m_ [Fig. 5B; ΔΨ_m_ decreased from 92.5±1.15 (control) to 84.4±1.13 and 77.38±1.63 a.u. (at 100 and 500 μM, respectively)] and by measuring cytoplasmic membrane phosphatidylserine translocation by Annexin V-FLUOS (Fig. 5A). The percentage of Annexin-positive cells in the population increased from 10.64±1.3 (control) to 19.6±2.5, 28.74±3.1 and 44.3±3.6 at 50, 100 and 500 μM, respectively.

## Discussion

Tumor cells are known to be resistant to apoptotic stimuli by several mechanisms. Tumors overexpress anti-apoptotic proteins such Bcl-2 and have decreased expression of pro-apoptotic proteins such as Bax or BH3 (21), have impaired signals from death receptors (22), or activated nuclear factor NF-κB which prevents activation of caspase-mediated cleavage (23). In our research, we showed that impaired calcium signals induced by capsaicin may result in ER stress and apoptosis. We observed significant calcium leakage from ER, which was associated with the overexpression of RyR2 calcium release channels. This depletion was apparently irreversible, as expression of sarco-endoplasmic ATPase, responsible for reload of calcium back to the ER, was substantially decreased. It is known that overexpression of RyR2 increases susceptibility of cells to apoptosis. For example, it was shown that overexpression of a splice variant of RyR2 may trigger apoptosis in the heart (24). SERCA2 has 3 binding sites for NF-κB in the promoter region and a decrease in expression may be explained by the inhibitory effect of capsaicin on NF-κB. It seems that capsaicin has a double impact on calcium homeostasis in the ER of PC12 cells. The first effect is caused by activation of the TRPV1 channel with possible changes in cationic fluxes. The second effect is modulation of the expression of calcium transporting systems RyR2 and SERCA2 with an impact on calcium content in the ER. Functional connection of these structures was recently described as TRPV1/RyR1 crosstalk in mouse skeletal muscle, where TRPV1 was expressed at ER membranes in the proximity of SERCA1 pumps (25). In our research, capsaicin was also found to modulate calcium rheostat proteins at the ER, and the Bcl-2/Bax ratio, which showed an expression shift to pro-apoptotic Bax. Several authors have shown that cells with overexpressed Bax, as well as cells having Bcl-2 deficiency, show decreased ER calcium content (7). Decrease in the expression of Bcl-2 protein caused by capsaicin is in accordance with our previous study (11), where we showed that capsaicin is an inhibitor of NF-κB. This transcription factor promotes upregulation of anti-apoptotic Bcl-2 (1).

ER stress-mediated apoptosis through the activation of CHOP has been intensively studied (26,27). CHOP (also called growth arrest DNA damage-inducible gene 153, GADD153) transcriptionally regulates genes that participate in the apoptotic pathway and it has been shown that increased levels of this protein are associated with inhibition of Bcl-2 which triggers the effect of the Bax/Bad systems in mitochondria (24). This effect may be antagonized by activation of NF-κB and upregulation of Bcl-2 (26,27). We also observed, in addition to an altered Bax/Bcl-2 ratio, elevated levels of CHOP suggesting that capsaicin modulates only this pathway. We also found enhanced expression of ATF4, which indicated an elevated ratio of more signals typical for ER stress, such as protein folding and degradation. Very similar results were obtained by Sánchez *et al* in prostate tumor cells. They showed by microarray, real-time PCR and western blotting techniques that capsaicin upregulates the CHOP and ATF4 pathways (28).

The pivotal role of IRE1α/XBP1 signaling in tumorigenicity has been well recognized (29). The spliced active form of XBP1 (XBP1s) acts as a transcription factor in the nucleus and activates genes for protein folding and restoration of ER homeostasis. In contrast, the unspliced form of XBP1 (XBP1u) functions as the dominant-negative form that antagonizes the function of XBP1s (30). We observed that capsaicin caused an increase in the XBP1 at ER. In connection to all these evidence for induced ER stress, we also found typical markers for apoptosis in the PC12 cells. Loss of mitochondrial membrane potential (ΔΨ_m_) and binding of Annexin V-FLUOS indicated that ER stress induced by capsaicin resulted in apoptosis. Taken together, capsaicin acts in PC12 cells by triggering ER stress in a concentration-dependent manner and this stress results in apoptosis.

This is the first study demonstrating that the primary signal induced by capsaicin is calcium release from the ER which consequently triggers a cascade of events leading to ERSR and apoptosis. These data may aid to the identification of new targets and pathways for cancer treatment.
